# Multicolor Fluorescence Imaging as a Candidate for Disease Detection in Plant Phenotyping

**DOI:** 10.3389/fpls.2016.01790

**Published:** 2016-12-02

**Authors:** María L. Pérez-Bueno, Mónica Pineda, Francisco M. Cabeza, Matilde Barón

**Affiliations:** Department of Biochemistry, Cellular and Molecular Biology of Plants, Estación Experimental del Zaidín – Spanish Council of Scientific ResearchGranada, Spain

**Keywords:** *Dickeya dadantii*, *Cucumis melo*, *Cucurbita pepo*, multicolor fluorescence imaging, thermal imaging, phenotyping, artificial neural network, logistic regression analysis

## Abstract

The negative impact of conventional farming on environment and human health make improvements on farming management mandatory. Imaging techniques are implemented in remote sensing for monitoring crop fields and plant phenotyping programs. The increasingly large size and complexity of the data obtained by these techniques, makes the implementation of powerful mathematical tools necessary in order to identify informative parameters and to apply them in precision agriculture. Multicolor fluorescence imaging is a useful approach for the study of plant defense responses to stress factors at bench scale. However, it has not been fully applied to plant phenotyping. This work evaluates the possible application of multicolor fluorescence imaging in combination with thermography for the particular case of zucchini plants affected by soft-rot, caused by *Dickeya dadantii*. Several statistical models -based on logistic regression analysis (LRA) and artificial neural networks (ANN)- were obtained for the experimental system zucchini-*D. dadantii*, which classify new samples as “healthy” or “infected.” The LRA worked best in identifying high dose-infiltrated leaves (in infiltrated and non-infiltrated areas) whereas ANN offered a higher accuracy at identifying low dose-infiltrated areas. To assess the applicability of these results to cucurbits in a more general way, these models were validated for melon infected by the same pathogen, achieving accurate predictions for the infiltrated areas. The values of accuracy achieved are comparable to those found in the literature for classifiers identifying other infections based on data obtained by different techniques. Thus, MCFI in combination with thermography prove useful at providing data at lab scale that can be analyzed by machine learning. This approach could be scaled up to be applied in plant phenotyping.

## Introduction

Plant pathogens are severe constraints to the production yield of crop fields worldwide. Current agricultural policies are aimed to minimize the use of pesticides and fertilizers through better targeting, and the integration with cultural control of weeds, pests, and diseases (Maloy, 2005). The implementation of precision agriculture relies on the development of technologies that allow the identification and mapping of constraints in the crop fields, such as imaging techniques (Mulla, 2013). They can be used to evaluate the effects of stress on plant metabolism (Cerovic et al., 1999; Barón et al., 2012, 2016). Consequently, imaging techniques are powerful non-destructive tools that have become essential: they provide crucial information for the decision-making and for the right timing of the procedures to be applied (Usha and Singh, 2013; Li et al., 2014; Mahlein, 2016).

Imaging techniques implemented on plant phenotyping provide complex and large scale spatial and temporal information, which is very difficult to analyze and interpret by conventional statistical methods. Another important contribution to precision agriculture is the development of mathematical tools that allow monitoring and classification of plants and fruits by the severity of the disease (Hahn, 2009), based on advanced statistical methods, as reviewed by Mulla (2013) and Behmann et al. (2015). Some of these mathematical tools could be used as classifiers, identifying stressed plants, or monitoring the evolution of pests. This strategy can also be applied in the same way on plant phenotyping programs (Fiorani and Schurr, 2013). The classifiers are mathematical models that are obtained by machine learning: systems that learn from data corresponding to different categories or subpopulations (Hahn, 2009; Behmann et al., 2015; Singh et al., 2016). Successful models are able to identify what category new data belong to, thus classifying them accurately. Machine learning includes a wide range of classifiers, such as ANN and LRA. ANN is a network inspired by biological neural networks that learn from input and output data (Hill et al., 1994). On the other hand, LRA is a statistical method that estimates the probability of a dichotomous outcome (“healthy” vs. “infected”) based on one or more independent variables. For this reason, LRA is of particular interest and widely used in biomedicine (Hosmer et al., 2013). Independently from the model used, part of the dataset obtained by experimental measurements (usually about two thirds of the total set of data) is used for training the model, and the remaining part is used for its validation. The goodness of the model is provided by the parameters sensitivity, specificity, and accuracy. The proportion of samples predicted to be infected that are actually “infected” is referred to as sensitivity or true positive rate, while the proportion of samples that are correctly predicted to be “healthy” is called specificity, or true negative rate. Accuracy is the proportion of right guesses, both “healthy” and “infected” samples (Parikh et al., 2008).

Reflectance and thermography are imaging techniques used widely in remote sensing and plant phenotyping. On the contrary, MCFI, otherwise very used in fundamental research on plant defense responses upon abiotic and biotic stress factors (Cerovic et al., 1999), has not been developed for its use at large scale. In the past, MCFI was applied in crop fields for some particular cases, although no systematic analysis of the images could be carried out at that time (Heisel et al., 1996; Johansson et al., 1996; Saito et al., 1998). In later years, advances have been made in its implementation at large scale (Tremblay et al., 2012; Latouche et al., 2015).

Multicolor fluorescence imaging is a non-invasive technique by which UV-excited autofluorescence is collected from plants or leaves. Fluorescence in the red and far red regions is emitted by chlorophyll *a*. In addition, the fluorescence in the blue and green regions (BGF) is emitted by secondary metabolites, many of them phenolics related to plant defense (Buschmann and Lichtenthaler, 1998). Therefore, the results serve as an indication of the activity of plant metabolism.

Imaging of leaf and canopy temperature by thermography has been widely used in plant phenotyping, mainly to characterize drought susceptibility (Li et al., 2014). Leaf temperature inversely correlates with transpiration and stomatal conductance (Jones, 1999), which is tightly regulated by plants as a general mechanism of defense upon abiotic stress, but also against pathogens (Melotto et al., 2008). This technique has been used in the study of infections by virus, bacteria, and fungi, as reviewed by Barón et al. (2016).

The pectinolytic *Dickeya* spp. are necrotrophic, Gram-negative plant pathogens that cause soft-rot disease and black-leg. A very wide range of plants are host for these species, including many economically important horticultural and ornamental plants worldwide (Czajkowski et al., 2011; Mansfield et al., 2012). *Dickeya dadantii* is able to infect fleshy, succulent plant parts, such as tubers, rhizomes, stems, and leaves, causing localized symptoms, thus limiting the crop yield and quality, and exerting significant losses in fields and in postharvest. *D. dadantii* is particularly pernicious due to its ability to live as saprophyte, epiphyte, or endophyte (Reverchon and Nasser, 2013), with great capacity for adaptation to new geographic areas and to new hosts (Reverchon et al., 2016). *D. dadantii* can be found in ground water, crop residues, soils, and also on other plants causing no infection, that would serve as a reservoirs (Nelson, 2009). It can also be isolated from the roots of healthy weeds in agricultural fields (Tsror et al., 2010). On the other hand, it can infect insects, which in turn may serve as dissemination vectors (Reverchon and Nasser, 2013). All these features make *D. dadantii* be among the top 10 most important bacterial pathogens in agriculture, according to Mansfield et al. (2012). In this scenario, early detection and appropriate farming practices are essential to minimize the spreading of infections. Bacteria enter primarily through hydathodes, stomata, and wounds to invade intercellular spaces. Once inside the plant tissue, *D. dadantii* produces and secretes degradative enzymes, mostly pectate lyases, which catalyze the hydrolysis of pectin, an essential component of the plant cell walls (Duprey et al., 2014). The consequent degradation of cell walls to gain access to nutrients, is the cause of soft-rot, the typical symptom of maceration (Hugouvieux-Cotte-Pattat et al., 1996).

In this work, the experimental host-pathogen systems were cucurbits (zucchini and melon) infected by *D. dadantii*. The main aim of this work is to explore the possible application of advanced statistical methods to data obtained by MCFI, on its own or by combination with thermography, for early disease detection. For this purpose, the predictions obtained by ANN and LRA are compared. The results show the convenience of MCFI in plant disease detection as a new approach for plant phenotyping.

## Materials and Methods

### Biological Material and Inoculation

Seeds of zucchini (*Cucurbita pepo*) v. Negro Belleza and melon (*Cucumis melo)* v. Rochet Panal (Semillas Fitó, Barcelona, Spain) were allowed to germinate in sterile conditions in petri dishes for 1 week at 24°C. Seedlings were planted in soil and transferred to a growth chamber under 150 μmol m^-2^ s^-1^ photosynthetically active radiation with a 16/8 h (22/18°C) light/dark photoperiod and 65% relative humidity.

*Dickeya dadantii* strain 3937, formerly *Erwinia chrysanthemi* 3937, was grown for 24 h at 28°C in Luria-Bertani (LB) plates containing 25 μg ml^-1^ rifampicin. Bacterial suspensions were prepared in 10 mM MgCl_2_ by adjusting their optical density at 600 nm to 0.1, which corresponded to 10^8^ colony forming units per ml. Serial dilutions of the bacterial suspension were carried out to obtain the two concentrations used for inoculations, high and low dose (HD and LD, 10^6^ or 10^4^ colony forming units per ml, respectively).

**FIGURE 1:**
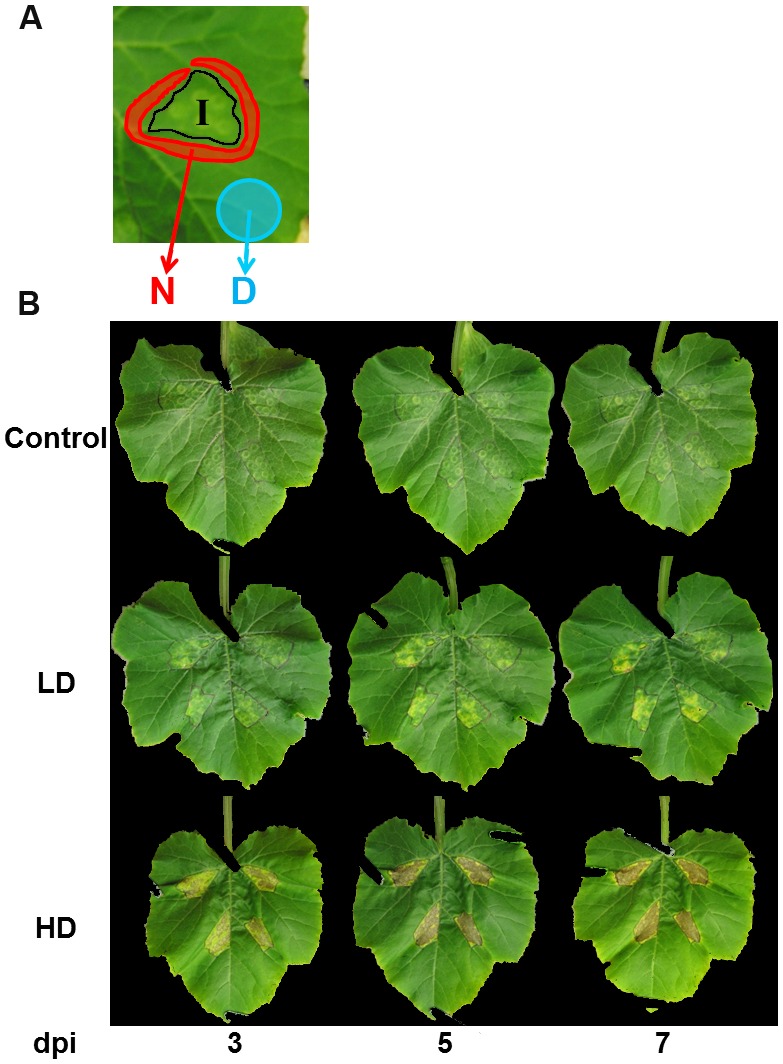
**(A)** Areas defined for image analysis of mock and bacteria-infiltrated zucchini leaves: I is the infiltrated area, accurately outlined using a marker pen at the moment of the infiltration; N is the neighboring area; and D are distal regions away from the I area. **(B)** Evolution of symptoms of zucchini leaves inoculated with *D. dadantii* at LD or HD.

The second leaf of 3-weeks old zucchini and melon plants was inoculated by infiltration as described in Pérez-Bueno et al. (2016) by pressing the LD or HD bacterial suspension into the abaxial side of the leaf using the blunt end of a 1 ml syringe. Mock-inoculated control plants were infiltrated with 10 mM MgCl_2_. Three regions of the leaf were defined: the infiltrated area (I, accurately outlined using a marker pen at the moment of the infiltration), neighboring area (N), and distant regions away from the I area (D), as shown in **Figure 1A** for zucchini. Infiltrations were carried out in four distant areas of approximately 1 mm^2^ on the second fully-developed leaf of each plant. Five plants per treatment and experiment were used. Seven independent experiments were carried out on zucchini, and measurements were taken at 3, 5, and 7 dpi, respectively. In the case of melon, four independent experiments were carried out and measurements were taken at 3 and 7 dpi.

### Leaf Thermography

Infrared images of plant leaves were taken in the growth chamber with a FLIR A305sc camera (FLIR Systems, Wilsonville, OR, USA) vertically positioned approximately 500 mm above the leaves according to Pérez-Bueno et al. (2015). The camera collects 320 × 240 pixel resolution images with a thermal sensitivity <0.05°C in the spectral range 7.5–13 μm. Thermal images were collected at midday over the period of study. Average temperatures were determined for the three leaf areas defined using the software FLIR Research & Development software version 3.4. Images, displayed using a false color scale, correspond to standard experiments.

### Autofluorescence by Multicolor Fluorescence Imaging

Multicolor fluorescence imaging was performed on the adaxial side of zucchini leaves using an Open FluorCam FC 800-O (Photon Systems Instruments, Brno, Czech Republic) according to Pérez-Bueno et al. (2015). Autofluorescence images were captured in the blue (F440), green (F520), red (F680), and far red (F740) regions of the spectrum. The fluorescence ratios F440/F520, F440/F680, F440/F740, F520/F680, F520/F740, and F680/F740 were also calculated. Black and white images of both fluorescence and calculated ratios were displayed using a false color scale, applied by the FluorCam software version 7.1.0.3. For each fluorescence parameter and ratio, average values were calculated for each of the three regions of interest, both for mock-control and bacteria-infiltrated leaves. Images correspond to standard experiments.

### Data Analysis

All images were analyzed considering the three defined areas (I, N, and D) separately. For each parameter, mean values were calculated per area and leaf using the software as described above. All calculations were performed with Microsoft Office Excel 2010 (Microsoft Corporation, Redmond, WA, USA). Statistical analysis of data was carried out using Student’s *t*-test with SigmaPlot 13.0v (Systat Software Inc., Richmond, CA, USA).

The large amount of data generated was classified in a database for each host-pathogen system using Microsoft Access 2010 (Microsoft Corporation). The database was used to train LRAs and ANNs based on multilayer perceptrons, a type of ANN, using R (R Core Team, Vienna, Austria). The learning heuristic used for training the ANNs was resilient backpropagation. The experimental parameters used to train the ANNs were those found more informative by the LRAs (**Tables 1** and **2**). The data obtained for around two thirds of all the zucchini samples analyzed was used for training (n specified in **Tables 1** and **2**). The rest, about 33% of the data, was used for validating the models. On the other hand, to evaluate their performance on melon samples, the zucchini models were validated with the whole dataset obtained for melon.

**Table 1:** Best logistic regression fits for the prediction of healthy and *Dickeya dadantii* infected zucchini leaves.

Area	dpi	Dose	*n*	Predictors	β coeff	*SE*	CI (95%)	*P* value	AIC
I	7	HD	172	Intercept	1,23	1,06	(-0,93–6,35)	0,209	-225,01
				F680/F740	8,14	2,16	(5,02–16,43)	0,000	
I	7	LD	172	Intercept	0,29	0,20	(-0,08–0,68)	0,131	-53,78
				F440/F520	-0,52	0,24	(-1,00–-0,06)	0,028	
				F440/F680	0,49	0,27	(-0,03–1,04)	0,065	
				F680/F740	1,42	0,29	(0,88–2,04)	0,000	
I	5	HD	128	Intercept	-0,52	0,99	(-4,89–1,49)	0,590	-161,65
				F520/F740	-17,00	6,99	(-60,62–-7,35)	0,000	
I	5	LD	128	Intercept	0,78	0,33	(0,18–1,5)	0,009	-48,86
				F440/F520	3,37	0,75	(2,06–5,06)	0,000	
I	3	HD	180	Intercept	1,64	0,56	(0,66–2,95)	0,000	-197,94
				T	0,65	0,19	(0,30–1,02)	0,000	
				F680	1,25	0,35	(0,61–1,98)	0,000	
				F440/F520	0,04	0,19	(-0,33–0,42)	0,819	
				F440/F740	1,16	0,36	(0,51–1,91)	0,000	
I	3	LD	180	Intercept	0,13	0,17	(-0,19–0,47)	0,422	-25,65
				F520	6,28	1,16	(4,34–9,17)	0,000	
N	7	HD	172	Intercept	0,20	0,21	(-0,21–0,62)	0,339	-82,91
				F440	1,69	0,35	(1,04–2,45)	0,000	
				F680	-0,91	0,25	(-1,43–-0,45)	0,000	
				F440/F520	0,13	0,24	(-0,33–0,6)	0,575	
				F680/F740	1,29	0,29	(0,76–1,9)	0,000	
N	7	LD	172	Intercept	0,05	0,15	(-0,25–0,34)	0,763	1,06
				F680	0,15	0,15	(-0,15–0,45)	0,333	
N	5	HD	128	Intercept	0,34	0,26	(-0,16–0,89)	0,183	-63,70
				T	0,56	0,26	(0,07–1,1)	0,024	
				F440	1,21	0,36	(0,56–1,98)	0,000	
				F520/F740	1,72	0,36	(1,09–2,51)	0,000	
N	5	LD	128	Intercept	0,00	0,18	(-0,35–0,35)	0,998	-4,25
				F440/F680	0,45	0,19	(0,10–0,83)	0,012	
N	3	HD	180	Intercept	0,15	0,19	(-0,22–0,53)	0,425	-69,48
				T	1,19	0,25	(0,73–1,72)	0,000	
				F440	1,42	0,29	(0,88–2,04)	0,000	
				F440/F520	-0,56	0,21	(-0,99–-0,15)	0,006	
				F680	1,14	0,29	(0,60–1,75)	0,000	
N	3	LD	180	Intercept	0,05	0,16	(-0,26–0,36)	0,745	-17,43
				T	0,52	0,20	(0,15–0,91)	0,006	
				F440	0,34	0,21	(-0,06–0,76)	0,100	
				F440/F520	-0,06	0,21	(-0,46–0,35)	0,783	
				F680/F740	0,75	0,22	(0,35–1,2)	0,000	
D	7	HD	172	Intercept	0,09	0,18	(-0,26–0,44)	0,615	-42,43
				T	0,68	0,19	(0,32–1,08)	0,000	
				F440	0,82	0,20	(0,45–1,24)	0,000	
				F680	-0,62	0,21	(-1,05–-0,22)	0,002	
				F680/F740	0,90	0,24	(0,46–1,39)	0,000	
D	7	LD	172	Intercept	0,05	0,16	(-0,26–0,35)	0,764	-3,54
				T	0,39	0,16	(0,07–0,71)	0,015	
				F520/F740	0,27	0,16	(-0,04–0,6)	0,084	
D	5	HD	128	Intercept	0,01	0,19	(-0,37–0,39)	0,970	-17,38
				T	0,69	0,21	(0,29–1,12)	0,000	
				F440/F520	0,01	0,21	(-0,39–0,42)	0,957	
				F440/F680	0,61	0,22	(0,20–1,06)	0,003	
D	5	LD	128	Intercept	0,00	0,18	(-0,35–0,35)	0,999	-0,16
				F680	-0,26	0,18	(-0,62–0,09)	0,141	
D	3	HD	180	Intercept	0,04	0,17	(-0,29–0,37)	0,810	-42,02
				T	1,15	0,20	(0,77–1,56)	0,000	
D	3	LD	180	Intercept	0,04	0,16	(-0,28–0,36)	0,800	-19,59
				T	0,51	0,20	(0,14–0,9)	0,007	
				F440	-0,46	0,23	(-0,95–-0,03)	0,035	
				F680	-6,55	2,50	(-11,94–-2,01)	0,003	
				f740	7,41	2,67	(2,55–13,16)	0,002	
				F440/F520	-0,77	0,28	(-1,34–13,16)	0,003	
				F680/F740	2,90	0,96	(2,55–13,16)	0,000	

*Area, leaf area; dpi, days post-inoculation; Dose, concentration of bacterial inoculum; n, sample size; Predictors, variables selected by the LRAs; β coeff, coefficients in the logistic function; SE, standard error; CI (95%), 95% confidence interval; P value, significance level; AIC, Akaike information criterion.*

**Table 2:** Artificial neural networks (ANN) models for the prediction of healthy and *D. dadantii* infected zucchini leaves.

Area	dpi	Dose	*n*	CE	Steps	AIC	BIC	Predictors	α1	β1	α2	β2
I	7	HD	172	0,02	97	14,04	36,07	F680/F740	-1,66	-9,41	1,68	9,16
I	7	LD	172	68,61	7141	159,22	193,84	F440/F520	-0,64	0,75	-36,64	49,01
								F440/F680		0,04		-25,09
								F680/F740		1,33		-17,71
I	5	HD	128	0,05	157	14,10	34,07	F520/F740	4,84	8,77	-4,46	-8,12
I	5	LD	128	67,12	5073	148,24	168,21	F440/F520	20,88	32,37	-91,41	93,77
I	3	HD	180	20,54	1705	55,07	77,42	T	-17,32	-52,90	18,77	-5,44
								F680		-76,26		-33,58
								F440/F520		-1,41		-8,35
								F440/F740		-59,67		-41,43
I	3	LD	180	90,27	4040	206,54	248,05	F520	4,53	-155,78	1,08	1,76
N	7	HD	172	57,44	3651	140,87	181,79	F440	-6,42	-1,59	21,44	56,65
								F680		1,23		-12,28
								F440/F520		1,93		35,35
								F680/F740		-1,47		4,08
N	7	LD	172	115,42	9241	244,85	266,88	F680	-30,70	26,38	-7,67	5,88
N	5	HD	128	37,01	3264	96,02	127,39	T	28,65	-25,42	43,90	0,87
								F440		-1,14		37,38
								F520/F740		-60,80		42,60
N	5	LD	128	81,15	1608	176,30	196,26	F440/F680	-91,26	-85,63	-24,12	12,12
N	3	HD	180	69,39	1764	164,77	206,28	T	1,66	-5,05	5,57	60,65
								F440		-10,05		17,59
								F440/F520		-8,93		-4,90
								F680		7,37		27,59
N	3	LD	180	101,70	10235	229,39	270,90	T	7,49	-45,20	2,12	-0,29
								F440		73,46		0,20
								F440/F520		97,05		0,08
								F680/F740		135,29		-0,90
D	7	HD	172	76,23	509	178,47	219,38	T	-0,24	-1,84	-1,32	-1,04
								F440		-4,14		-2,22
								F680		8,67		2,05
								F680/F740		-0,46		-0,83
D	7	LD	172	103,88	1892	225,77	254,09	T	32,09	-27,19	-33,71	-15,51
								F520/F740		4,36		-74,92
D	5	HD	128	64,66	3710	151,33	182,70	T	27,18	-69,85	-63,79	0,33
								F440/F520		9,90		-26,73
								F440/F680		-63,79		-52,54
D	5	LD	128	86,22	2079	186,44	206,41	F680	22,01	-30,16	-22,84	7,68
D	3	HD	180	101,14	7110	216,28	238,63	T	-0,48	-0,56	76,82	46,41
D	3	LD	180	92,36	23597	218,73	273,01	T	461,05	330,73	-7,96	25,88
								F440		362,46		-6,06
								F680		21,13		-81,52
								F740		0,19		105,41
								F440/F520		-73,28		-29,74
								F680/F740		-289,81		26,89

*Area, leaf area; dpi, days post-inoculation; Dose, concentration in bacterial inoculum; n, sample size; CE, cross-entropy; Steps, number of learning steps; AIC, Akaike information criterion; BIC, Bayesian information criterion; Predictors, independent variables selected by the LRAs; α1 and α2, intercept of hidden neurons 1 and 2, respectively; β1 and β2, slope of hidden neurons 1 and 2, respectively. In all cases, all the ANNs had one hidden layer with two neurons, epoch was five and threshold was 0.01*.

## Results

### Symptomatology in Zucchini

The symptoms of infection by *D. dadantii* in the three regions of interest (**Figure 1A**) are shown in **Figure 1B**. In the case of LD-infiltration, symptoms consisted in chlorosis that developed progressively from 3 dpi in the I area, followed by the appearance of small necrotic spots from 7 dpi. N and D areas of LD-infiltrated leaves showed no symptoms throughout the period of study.

In the case of HD-infiltrated leaves, I areas showed signs of maceration within few hours after infiltration, leading to death of the infiltrated tissue by 3 dpi. N and D areas of HD-infiltrated leaves developed chlorosis progressively from 7 dpi.

### Effect of the Infection on Secondary Metabolism of Zucchini Leaves

**FIGURE 2:**
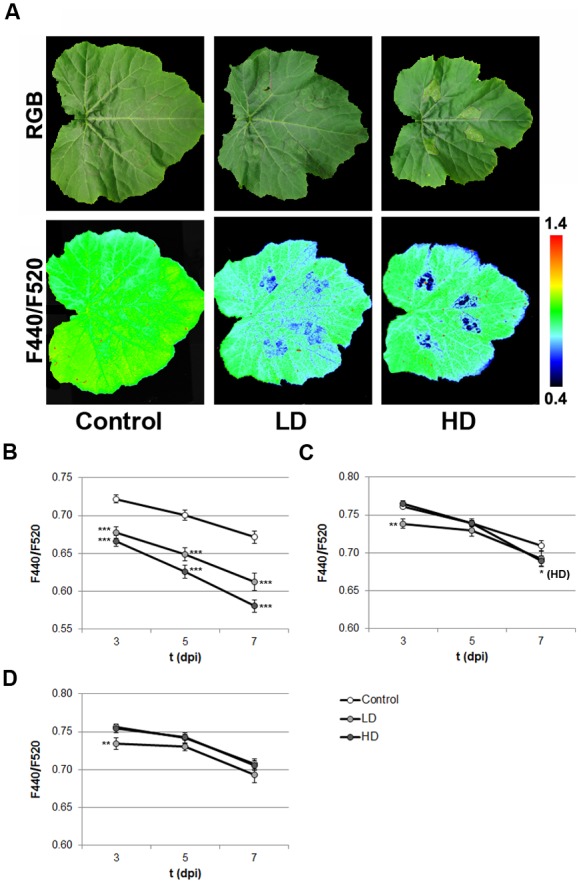
**(A)** Standard F440/F520 images of mock-control, LD, and HD-inoculated zucchini leaves at 3 dpi, and their corresponding RGB images. Progression of F440/F520 signal in I **(B)**, N **(C)**, and D **(D)** areas of the leaves along the period of study. Symbols ^∗^, ^∗∗^, and ^∗∗∗^ stand for *p* < 0.1, 0.01, and 0.001, respectively.

The infection by *D. dadantii* caused an increase in the BGF. This increase was restricted to the I areas, and was statistically significant from 3 or 5 dpi in HD or LD infected leaves, respectively (data not shown). However, the ratios F440/F740, F520/F740, and particularly F440/F520 (**Figure 2A**), showed changes in the LD leaves prior to the development of symptoms. The value of F440/F520 significantly decreased (*p* < 0.001) in I areas of LD and HD-infiltrated leaves from 3 dpi (**Figure 2B**), proportionally to the time post-infection and bacterial dose. On the other hand, the N and D areas of LD infected leaves showed statistically significant decreases in F440/F520 at 3 dpi (*p* < 0.01). In contrast, among the non-infiltrated areas of HD leaves, only N areas showed significant differences at 7 dpi (*p* < 0.1; **Figures 2C,D**).

### Effect of the Infection on Zucchini Leaf Transpiration

**FIGURE 3:**
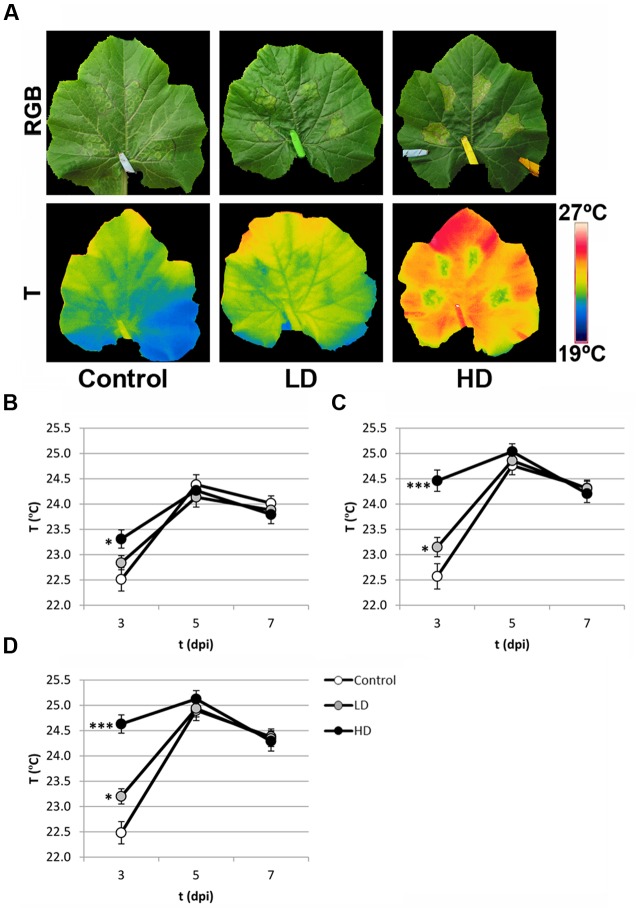
**(A)** Standard thermal images of mock-control, LD, and HD-inoculated zucchini leaves at 3 dpi, and their corresponding RGB images. **(B)** Evolution of temperature in I **(B)**, N **(C)**, and D **(D)** areas of the leaves along the period of study. Symbols ^∗^ and ^∗∗∗^ stand for *p* < 0.1 and 0.001, respectively.

At earlier stages of the infection (3 dpi), the infection by *D. dadantii* caused an increase in the temperature across the whole inoculated leaves (**Figure 3A**). In I areas, this increment was only significant for HD at 3 dpi (0.7°C, *p* < 0.1; **Figure 3B**). Conversely, the most drastic increase was found in the N and D areas of HD inoculated leaves, where the temperature increased up to 2°C relative to the temperature of corresponding areas in mock-control leaves (*p* < 0.001). Moreover, the temperature in the N and D areas of LD leaves also increased, although to a lesser extent (0.6–0.7°C, *p* < 0.1; **Figures 3C,D**). Later in the infection process the temperature of inoculated leaves decreased reaching control values.

### Statistical Models for Diagnosis on Zucchini Infected Plants

Prior to the development of the first symptoms, autofluorescence and thermography images did not report a pattern that could be clearly correlated to the infection. However, the numeric data obtained from these images could be analyzed using mathematical tools to build statistical models. The values for all the MCFI parameters measured (F440, F520, F680, and F740) and those calculated (F440/F520, F440/F680, F440/F740, F520/F680, F520/F740, and F680/F740), plus the temperature were arranged on a database by leaf area and dpi. Thus, the data could be used to fit a LRA per leaf area, dpi and bacterial dose. In this way, the predictors, informative variables offering a clear contrast between healthy and infected leaves, were determined. The predictors that provided the best fits by LRA for each leaf area and dpi are shown in **Table 1**. According to the Akaike information criterion (AIC) and the 95% confidence interval (CI) provided in **Table 1**, the best fits were those for I areas, followed by those for N areas. The fits for HD leaves were in general better than those for LD leaves.

**FIGURE 4:**
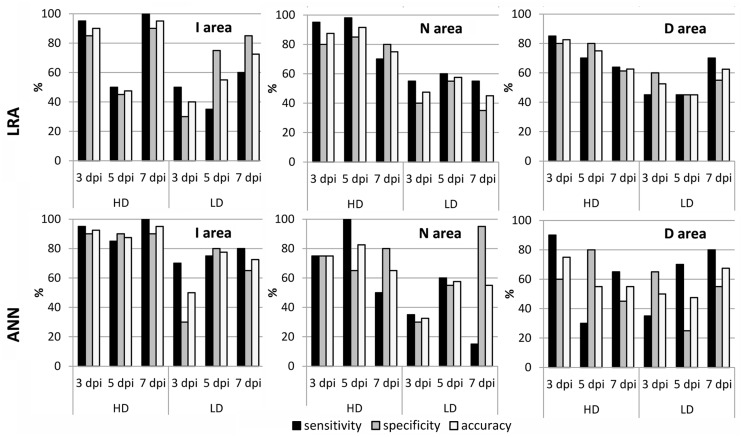
**Sensitivity, specificity, and accuracy of the best fits obtained by LRA and for the best ANN for the I, N, and D areas of zucchini leaves LD and HD-inoculated with *D. dadantii***.

The goodness of the fits obtained by LRAs and ANNs was evaluated for zucchini plants infected by *D. dadantii* with new data from samples previously not used for training the models (**Figure 4**). In the overall, the models with the highest accuracy (90–100%) were those ANNs obtained for I areas of HD-infected leaves. On the contrary, the LRAs showed higher specificity and sensitivity than the ANNs when classifying N and D areas. For HD-infected zucchini leaves, the LRAs of N areas showed an accuracy ranging from 75 to 92% and for those of D areas the accuracy was 63–83%. In the case of LD-infected leaves, the models with the highest accuracy (75–80%) were the ANNs for I areas, especially at 5 and 7 dpi.

### Effect of the Infection on the Metabolism of Melon Leaves and Applicability of Zucchini-*D. dadantii* Statistical Models

The effect of soft-rot was assessed on another cucurbit, *Cucumis melo* (melon), inoculated with *D. dadantii* following the same experimental design described above for zucchini. Symptoms were similar to those described for *D. dadantii*-zucchini infected plants. Moreover, the metabolic changes in melon leaves upon infection were similar to those found in zucchini.

**FIGURE 5:**
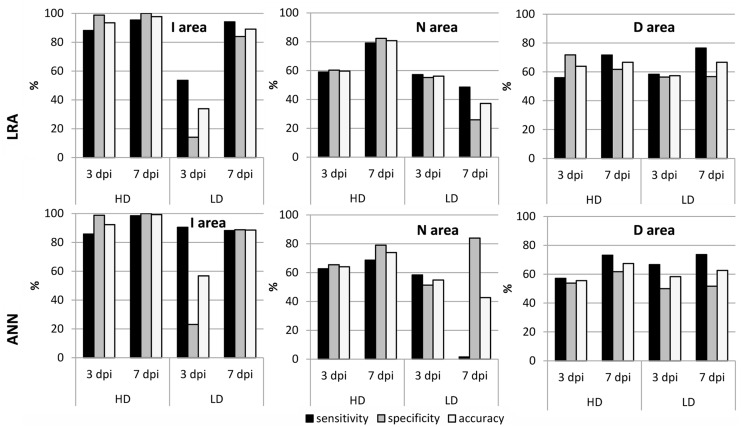
**Sensitivity, specificity, and accuracy of the zucchini models applied to melon.** The best fits obtained for zucchini (by LRA and ANN) were applied to the corresponding areas of melon leaves LD and HD-inoculated with *D. dadantii*.

The suitability of the prediction models generated for zucchini leaves was assessed for melon by validating the models with the dataset obtained for *D. dadantii*-infected melon plants. The performance of zucchini models on melon plants infected at HD was very similar to that found on zucchini plants infected with LD. The prediction models performed best for I areas, for which both LRAs and ANNs had values of sensitivity and specificity of 80–100%, providing a very high accuracy of 95–100%. However, these values were 50–70% in N and D areas (**Figure 5**).

## Discussion

Dealing with current problems in agriculture involves the development of new methodology to evaluate and monitor crops. Li et al. (2014) and von Bueren et al. (2015) have reviewed a number of techniques that are currently in use in vegetation analyses, including several techniques based on reflectance (RGB imaging, hyperspectral near infrared, multi and hyperspectral spectrometers), and thermography. Reflectance and thermography are well established approaches in precision agriculture (Oerke et al., 2014; Humplik et al., 2015). These techniques have also been applied to automated high-throughput analysis for plant phenotyping. In most cases, the screenings aimed for varieties resistant mainly to abiotic stress factors such as drought, heat and cold, salt stress, and nutrient deficiency, as reviewed by Humplik et al. (2015). A relatively small number of works have addressed the study of biotic stress, most of them focused on fungal infections. For example, Baranowski et al. (2015) reported the analysis of *Alternaria* infections in oilseed rape; Calderón et al. (2013, 2015) analyzed olive trees infected by *Verticillium dahliae*; the infection of almond trees with red leaf blotch was studied by López-López et al. (2016); and powdery mildew on tomato plants was investigated by Raza et al. (2015). However, other techniques on their own, or combined, could also be of great help in precision agriculture and plant phenotyping, as reviewed by Tremblay et al. (2012).

Thermography provides useful information in the study of biotic stress. It reflects changes in leaves temperature as a consecuence of modifications in the stomatal aperture, which regulates evapotranspiration (Jones, 1999). The activation of stomatal closure triggered by recognition of pathogen-associated molecular patterns is a widespread defense mechanism in vascular plants against bacterial invasion via abscisic acid, salicylic acid, and jasmonic acid (Melotto et al., 2006, 2008; Sawinski et al., 2013). The increase of temperature found at 3 dpi in zucchini seemed to be dose-dependent. It is worth noticing that the larger differences relative to mock-control values were found in N and D regions of the leaves, where no symptoms were visible (**Figure 1**). This is in agreement with Pérez-Bueno et al. (2016).

Multicolor fluorescence imaging has offered several parameters as good markers of infection in the zucchini-*D. dadantii* system. A vast number of phenolic compounds produced by the plant secondary metabolism are part of the defense response. These compounds emit BGF upon excitation with UV light (Cerovic et al., 1999; Dixon, 2001). According to the findings here reported, F440 and F520 of I areas significantly increased when compared to the mock-controls at 3 (HD) or 5 dpi (LD). Both F440 and F520 also increased in N and D areas of infected leaves. These results point to an enhancement on plant secondary metabolism in response to infection (Buschmann and Lichtenthaler, 1998; Cerovic et al., 1999). Moreover, F520 increased to a higher extent than F440, causing the decrease in their ratio F440/F520. This ratio showed statistically significant differences at 3 dpi in the three regions of interest. At later timepoints, the same trend was observed although no significant differences could be found out of the I areas. However, these results were consistent in all the experiments carried out. A decrease in F440/F520 values have been previously reported in relation to long-term stress conditions, under which the accumulation of particular green fluorescing compounds could be induced (Buschmann and Lichtenthaler, 1998).

For a long time, MCFI has been used in fundamental research to study the effects of a variety of stress factors in plant metabolism. BGF is well known to be influenced by the nutrient content in soil. Several authors reported changes in BGF related to alterations in the phenolics content in plants upon treatments with low or high nitrogen, depending on the species (Heisel et al., 1996; Langsdorf et al., 2000). MCFI has also been used in a number of works analyzing the effect of pathogens in their hosts. Viral infections in model plants have been analyzed by Pineda et al. (2008) and in crop plants by Chaerle et al. (2007) and Montero et al. (2016). Some bacterial and fungal infections have been studied by this technique (Granum et al., 2015; Pérez-Bueno et al., 2015, 2016). Currently, the use of this technique on proximal sensing is limited to the use of few available devices (Cerovic et al., 2012; Latouche et al., 2015). These devices have proved useful in the assessment of flavonoids content in grapes or kiwifruit, and in the detection of the nitrogen status for several species (Julkunen-Tiitto et al., 2014). However, the devices available up to date are not imaging sensors, which limits their applicability in field measurements and plant phenotyping programs. To our best knowledge, this work is the first one using data provided by MCFI in combination with thermal imaging to obtain statistical models able to identify infected plants at lab scale.

Data obtained from healthy and infected zucchini leaves by MCFI and thermography were used to train LRAs and ANNs. These models were validated for zucchini healthy and infected samples, achieving in some cases high values of accuracy, particularly for I areas. It is worth noticing that zucchini models also showed a very high accuracy for the classification of I areas of melon leaves. Other authors have addressed the detection of other infections by statistical models. Thus, infection by huanglongbing in citrus could be detected by support vector machine classification trees (Sankaran et al., 2013) and blight diseases on tomato leaves by extreme learning machine (Xie et al., 2015) with an accuracy ranging from 70 to 100%, respectively. On the other hand, several diseases in cucurbits have been analyzed by a combination of thermal, chlorophyll fluorescence dynamics, and hyperspectral imaging (Berdugo et al., 2014). In that work, a general linear model was able to classify plants infected with two different viruses and one fungal pathogen with an accuracy of 85–100%. Moreover, Rumpf et al. (2010) reported a support vector machine classifying beetroot leaves infected with three different fungi with an accuracy of 65–100%, based on measurements with a non-imaging spectroradiometer. The accuracy of the models in this work is comparable to those found in the literature, although none of them made use of MCFI.

The results here reported show the potential application of MCFI, in combination with thermography, particularly to classify infiltrated (symptomatic) areas as “healthy” or “infected.” The automatic detection of symptomatic areas has been carried out by other authors using image processing (Al-Hiary et al., 2011; Tian et al., 2012; Pujari et al., 2015). This pre-analysis of images was applied by Huang (2007) to isolate symptomatic areas caused by bacterial diseases in *Phalaenopsis* seedlings prior to their classification by mathematical models. Such an approach could constitute a possible strategy to scale up the use of MCFI to crop fields or phenotyping. Further development of the technique would be desirable in order to facilitate its applicability in plant phenotyping and breeding programs.

## Author Contributions

MP-B and MP took part in the experimental design, acquisition, analysis, and interpretation of data, and on writing up. FC contributed to the data analysis. MB took part in the experimental design, the interpretation of data and writing up.

## Conflict of Interest Statement

The authors declare that the research was conducted in the absence of any commercial or financial relationships that could be construed as a potential conflict of interest.
